# Identifying villages and breeding habitats for dengue transmission in Thailand: insights from long-term larval surveys

**DOI:** 10.1186/s12879-024-09398-7

**Published:** 2024-05-24

**Authors:** Naiyana Sahavechaphan, Asamaporn Chatrattikorn, Manot Rattananen, Pongsakorn Sadakorn, Darin Areechokchai, Sopon Iamsirithaworn

**Affiliations:** 1 National Electronics and Computer Technology Center, National Science and Technology Development Agency, 112 Thailand Science Park, Phahonyothin Road, 12120 Pathum Thani, Thailand; 2 Department of Disease Control, Ministry of Public Health, 88/21 Tawanon Road, 11000 Nonthaburi, Thailand; 3 Ministry of Public Health, 88/20 Tawanon Road, 11000 Nonthaburi, Thailand

**Keywords:** *Aedes* mosquito, Dengue fever, TanRabad, Visual larval survey

## Abstract

**Background:**

In Thailand, the Department of Disease Control (DDC) regularly performs visual larval surveys throughout the country to monitor dengue fever outbreaks. Since 2016, the DDC switched from a paper-based to a digital-based larval survey process. The significant amount of larval survey data collected digitally presents a valuable opportunity to precisely identify the villages and breeding habitats that are vulnerable to dengue transmission.

**Methods:**

The study used digitally collected larval survey data from 2017 to 2019. It employed larval indices to evaluate the risk of dengue transmission in villages based on seasonal, regional, and categorical perspectives. Furthermore, the study comprehensively scrutinized each container category by employing different measures to determine its breeding preference ratio.

**Results:**

The result showed that villages with a very high-risk of dengue transmission were present year-round in all regions, with the highest proportion during the rainy season. The Southern region had more high-risk villages during the winter season due to rainfall. Slums and residential communities were more vulnerable to dengue than commercial areas. All container categories could potentially serve as breeding habitats for dengue-carrying mosquitoes, with abandoned containers being the most significant breeding sites.

**Conclusions:**

The risk of dengue transmission was present year-round throughout Thailand. This underscores the importance of community and government initiatives, along with sustained public awareness campaigns and active community engagement, to efficiently and permanently eradicate mosquito breeding habitats. It should be noted that larval indices may not strongly correlate with dengue cases, as indicated by the preliminary analysis. However, they offer valuable insights into potential breeding sites for targeted preventive measures.

**Supplementary Information:**

The online version contains supplementary material available at 10.1186/s12879-024-09398-7.

## Background

Dengue fever has been a significant public health concern globally in regions with tropical and sub-tropical climates [1–3]. The number of reported dengue cases has increased drastically over the years, from 505,430 cases in 2000 to over 2.4 million in 2010 and 5.2 million in 2019. During the period between 2000 and 2015, the number of reported deaths due to dengue fever increased from 960 to 4,032. The first dengue fever cases in Thailand were reported in 1949. Over the last 60 years, the country has experienced a surge in dengue fever outbreaks, with over 50,000 cases reported annually [4–6]. In a pandemic year, more than 100,000 cases are reported, and 100 or more deaths occur. The epidemic patterns vary, including every other year, two years apart, and a two-year epidemic with one- or two-year intervals. Most of the epidemic-prone areas are urban due to population movement and density and the increasing number of wet containers.

Dengue fever is primarily transmitted through the bites of female *Aedes* mosquitoes [7, 8]. These mosquitoes contract the dengue virus by feeding on the blood of infected persons. Once infected, the mosquitoes can transmit the virus to healthy individuals through their bite and also to their eggs. Mosquitoes breed in a variety of natural and artificial water-holding containers [9–11]. These containers can produce many larvae and pupae even with low rainfall [12]. Therefore, the prevention and control of dengue fever outbreaks relies on limiting contact between people and mosquitoes and eliminating potential breeding containers [13–19].

The visual larval survey is a critical tool for monitoring dengue fever outbreaks. In Thailand, the Department of Disease Control (DDC) under the Ministry of Public Health recognizes its importance and conducts regular surveys throughout the country. Since 2016, the DDC has been using the TanRabad SURVEY mobile application [20, 21] to transition from a paper-based to a digital-based larval survey process. Accordingly, this study aims to analyze the extensive larval survey data available in the TanRabad database and identify the villages and breeding habitats that contribute to the transmission of dengue.

The findings showed that villages with a very high-risk of dengue transmission were present year-round in all regions, with the highest proportion observed during the rainy season. The Southern region also had a greater proportion of very high-risk villages during the winter season, as the region experienced rainfall during such season. Among the three village categories, slums and residential communities were more vulnerable to dengue transmission, with a significantly higher House Index than commercial areas. The most significant breeding habitats for dengue-carrying mosquitoes were abandoned containers, which were prevalent throughout all seasons, all village categories, and all cultural regions. However, regularly used containers, such as anti-ant bowls, drip trays of water dispenser and other used containers, were also found to be essential breeding grounds. These findings emphasize the need for a sustained public awareness campaign and active community engagement to efficiently and permanently eradicate mosquito breeding habitats.

## Methods

### Study area

**Fig. 1:**
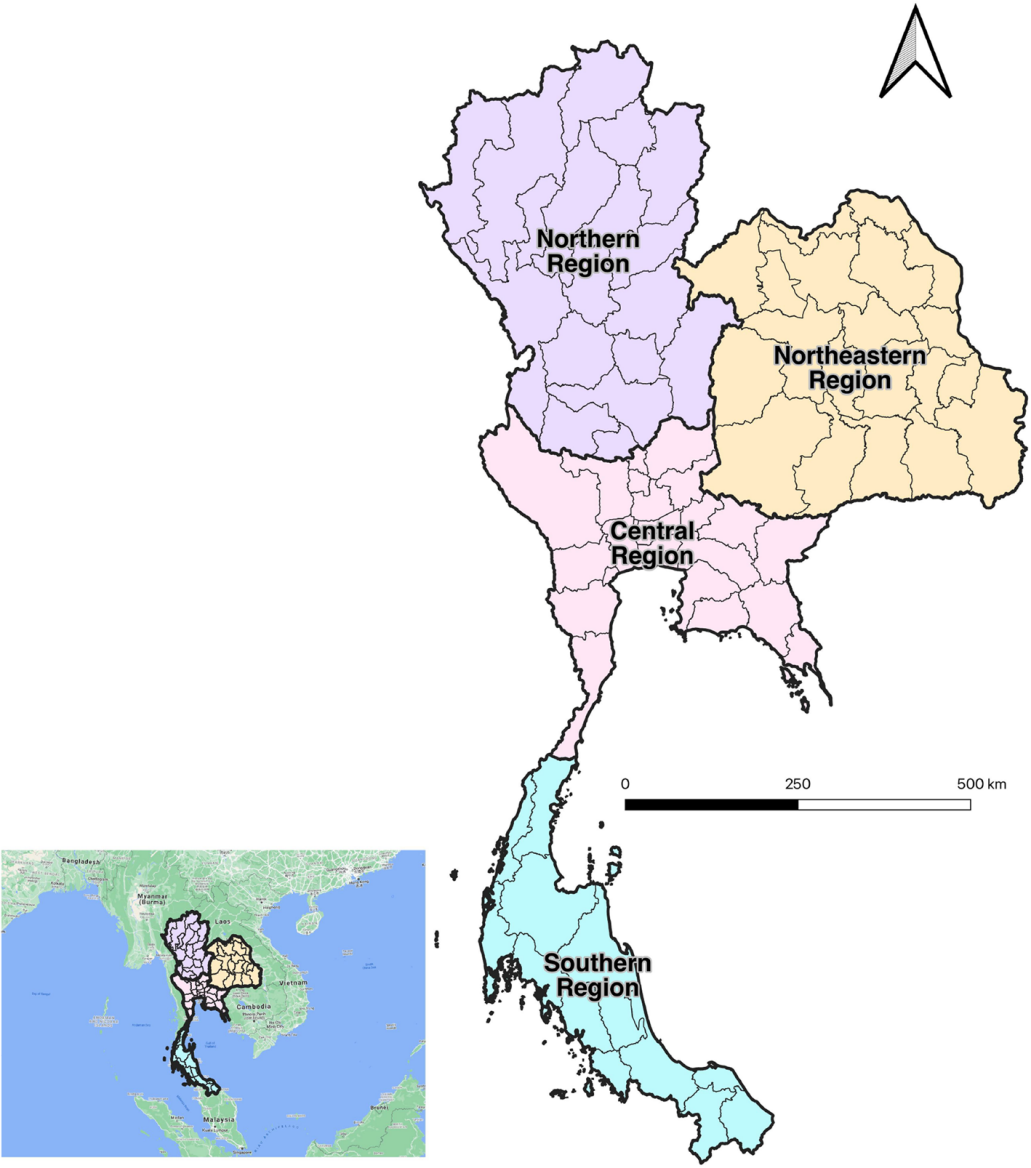
Study area (The bold and light boundaries indicate cultural regions and provinces, respectively)

Thailand is located at the centre of the Indochinese Peninsula and roughly between 5.61^∘^ N to 20.45^∘^ N in latitude and 97.37^∘^ E to 105.62^∘^ E in longitude. Thailand is administratively divided into 77 provinces and four cultural regions, as shown in Fig. 1, covering an area of 513,120 square kilometres and a population of over 70 million people. Thailand experiences a tropical climate influenced by seasonal monsoon winds [22]. The Southwest monsoon brings widespread rainfall across the country, while the Northeast monsoon brings cold and dry air over many parts. Thailand has three official seasons [23] - summer, rainy, and winter. The summer season runs from mid-February through to mid-May, with April and May the hottest months of the year, the rainy season is from mid-May to mid-October, and the winter season is between mid-October and mid-February. Mean annual rainfall ranges from 1,200 to 4,500 mm, with lower totals on the leeward side and higher totals on the windward side. The mean temperature ranges from 18 to 38^∘^C.

### Data source

**Fig. 2:**
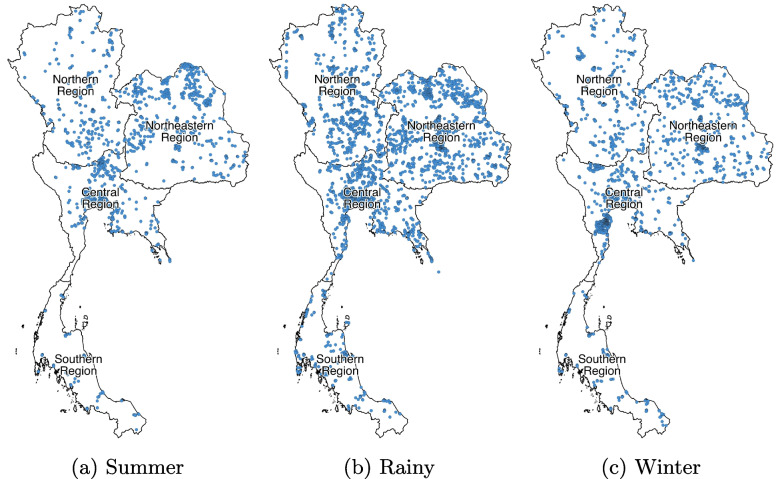
Spatial distribution of inspected villages

This study relied on the larval survey data spatially and temporally collected from 2017 to 2019 nationwide by public health officials of DDC using a mobile application TanRabad SURVEY [20, 21]. DDC has adapted the larval survey guideline from the World Health Organization (WHO) [14, 24–26] to align with Thai culture and tradition. According to DDC’s protocol, public health officials are required to inspect a random selection of villages classified as slums, residential communities and commercial communities (see Table 1) every three months per an urban district and every month per an at-risk district annually predicted. An at-risk district is defined as one where the number of cases in the past four weeks is greater than the average number of cases during the same four weeks in the previous five years. During the larval survey, officials examine at least 100 randomly selected houses in each village and report the total number of wet containers inspected and infested by each category (see Table 2) as well as each location (indoor and outdoor) in each house. In addition, when there is a report of dengue cases, public health officials are required to inspect their villages within a day after. Table 3 illustrates the seasonal number of inspected and infested containers for 9, 910 villages inspected nationwide in 2017-2019 (1, 786, 4, 978, and 3, 146 villages for summer, rainy, and winter seasons, respectively). The spatial distribution of inspected villages is shown in Fig. 2.

**Table 1:** Village categories

Village categories	Description
Slum	A village with a high population density and a lack
	of adequate living conditions.
Residential Community	A village designed for individuals or families to live in
	with appropriate management in place.
Commercial Community	A village consists of both residential and commercial
	buildings, with more than 40% of the buildings
	designated for commercial purposes.

**Table 2:** Container categories

Container categories	Description
**Artificial Containers** (used)	
Water Tank	A container used for various purposes, usually made
	of stone or earthenware, and featuring a large
	opening. Some examples are for taking a shower
	and washing the dishes.
Water Drinking Jar	A container used for drinking purposes, usually
	made of glass, stone or earthenware, and featuring
	a small opening and/or spout.
Vase	A container with decorative purposes, usually made
	of glass or china, that can be used as an ornament
	or for displaying cut flowers.
Lotus Basin	A capacious circular container with an open top
	designed for cultivating aquatic plants.
Pet Bowl	A deep, circular dish or basin utilized to serve food
	or liquids to pets.
Plant Saucer	A shallow receptacle placed beneath a container
	used for planting, intended to collect excess water
	that drains out.
Anti-ant Bowl	A container with a raised rim design that prevents
	ants from crawling into the bowl.
Drip Tray of Water Dispenser	A receptacle designed for carrying water from
	dispensers or refrigerators.
Other Used Container	Containers not included in the aforementioned
	categories and employed for various other purposes.
**Artificial Containers** (abandoned)	
Old Tire	An unused rubber casing, often filled with air or
	enclosing an inner tube that is inflated, fitted onto
	a wheel to create a flexible connection with the
	surface of the road.
Unused Container	Discarded containers such as fractured cans and
	unused bottles.
**Natural Containers**	
Plant Leaf	A flattened plant part that is directly or indirectly
	connected to the stem by a stalk.

**Table 3:** The number of wet containers inspected and infested from 2017 to 2019 by seasons (unit: pieces)

Container categories	Seasons						Total	
	Summer		Rainy		Winter			
	Inspected	Infested	Inspected	Infested	Inspected	Infested	Inspected	Infested
**Artificial Containers** (used)								
Water Tank	166,074	10,369	528,488	31,362	345,092	13,873	1,039,654	55,604
Water Drinking Jar	24,571	377	87,932	1,083	94,770	1,034	207,273	2,494
Vase	16,757	348	63,647	1,796	45,613	819	126,017	2,963
Lotus Basin	18,168	443	49,027	1,492	29,342	644	96,537	2,579
Pet Bowl	9,546	335	28,318	2,303	27,257	711	65,121	3,349
Plant Saucer	5,654	262	17,071	1,216	13,318	551	36,043	2,029
Anti-ant Bowl	4,473	427	14,379	1,435	13,321	797	32,173	2,659
Drip Tray of Water Dispenser	2,800	185	10,433	897	7,316	362	20,549	1,444
Other Used Container	15,668	1,049	62,025	6,304	37,698	1,662	115,391	9,015
**Artificial Containers** (abandoned)								
Old Tire	4,886	382	27,581	4,349	14,030	749	46,497	5,480
Unused Container	11,915	971	47,754	6,955	30,022	2,029	89,691	9,955
**Natural Containers**								
Plant Leaf	1,562	55	9,772	305	8,115	185	19,449	545
**Total**	**282,074**	**15,203**	**946,427**	**59,497**	**665,894**	**23,416**	**1,894,395**	**98,116**

### Data analytics

#### Villages as harbor of breeding habitats

Larval indices [27, 28] are calculated per an inspected village. These larval indices are (i) House Index (HI) – a percentage of houses infested with larvae or pupae; and (ii) Container Index (CI) – a percentage of wet containers infested with larvae or pupae. They are formally defined in the following formulas.

$$\begin{aligned} HI = \frac{Number\; of\; infested\; houses}{Number\; of\; inspected\; houses} \times 100 \end{aligned}$$

$$\begin{aligned} CI = \frac{Number\; of\; infested\; containers}{Number\; of\; inspected\; containers} \times 100 \end{aligned}$$

DDC has classified the risk level of dengue transmission into four categories based on the House Index (HI) and Container Index (CI), as outlined in Table 4: safe, low, moderate, and high [29–31]. These indices can be combined to further classify the dengue transmission risk levels of villages (or village risk levels for short) into ten different classes, ranging from the safe class (S$\times$S) to the very high-risk class (H$\times$H), as shown in Table 5. A safe village is free from dengue transmission risk, while a very high-risk village has a high transmission risk that is spread across many households. Moreover, a village risk level M$\times$H indicates a high transmission risk within some clusters of a village.

**Table 4:** Dengue transmission risk levels

Transmission risk levels	House index (HI)	Container index (CI)
Safe	0.0	0.0
Low	$0.1 - 10.0$	$0.1 - 5.0$
Moderate	$10.1 - 20.0$	$5.1 - 10.0$
High	$> 20.0$	$> 10.0$

**Table 5:** Dengue transmission risk levels for villages

HI-based risk levels	CI-based risk levels	Village risk levels
Safe	Safe	S$\times$S (safe)
Low	Low	L$\times$L (low-risk)
Low	Moderate	L$\times$M
Low	High	L$\times$H
Moderate	Low	M$\times$L
Moderate	Moderate	M$\times$M (moderate-risk)
Moderate	High	M$\times$H
High	Low	H$\times$L
High	Moderate	H$\times$M
High	High	H$\times$H (very high-risk)

#### Containers as breeding habitats

The breeding preference ratio (BPR) [12], the ratio of breeding contribution (BC) to breeding potentiality (BP), is calculated for each container category. A specific container category *c* is deemed essential if its BPR is greater than 1.0. It is formally defined for a particular container category *c* using the following formulas:

$$\begin{aligned} BPR = \frac{BC}{BP} \end{aligned}$$

$$\begin{aligned} BC = \frac{Number\; of\; infested\; containers\; with\; category\; c}{Number\; of\; infested\; containers} \times 100 \end{aligned}$$

$$\begin{aligned} BP = \frac{Number\; of\; inspected\; containers\; with\; category\; c}{Number\; of\; inspected\; containers} \times 100 \end{aligned}$$

#### Objective and hypotheses

The primary aim of this study is to pinpoint villages and breeding habitats contributing to dengue transmission using larval indices and the breeding preference ratio (BPR). We have three research questions with three pairs of null/alternative hypotheses as follows:

- Are larval indices consistent between any two seasons? H_0_: there is no significant difference H_1_: there is a significant difference
- Are larval indices consistent between any two village categories? H_0_: there is no significant difference H_1_: there is a significant difference
- Are larval indices consistent between any two cultural regions? H_0_: there is no significant difference H_1_: there is a significant difference

## Results

### Overall perspective

Table 6 presents the distribution of village risk levels across the country during the three seasons. Each season had ten village risk levels, which were differentiated based on a specific characteristic. During the summer season, the most frequent village risk levels were safe (23.80%), low-risk (21.00%), and very high-risk (17.19%). In the rainy season, the top three village risk levels were very high-risk (25.29%), safe (19.10%), and low-risk (17.92%). In the winter season, the three most prevalent village risk levels were safe (31.18%), low-risk (19.20%), and very high-risk (14.78%). Importantly, very high-risk level was among the top three village risk levels in all seasons. Moreover, villages in the rainy season had experienced a higher proportion of very high-risk level compared to the summer and winter seasons.

**Table 6:** Percentage of village risk levels across three seasons

Village risk levels	Seasons (%)		
	Summer	Rainy	Winter
S$\times$S (safe)	23.80	19.10	31.18
L$\times$L (low-risk)	21.00	17.92	19.20
L$\times$M	13.44	11.09	12.17
L$\times$H	2.58	4.48	5.12
M$\times$L	1.40	0.54	0.70
M$\times$M (moderate-risk)	8.29	6.77	5.79
M$\times$H	10.69	13.74	9.98
H$\times$L	0.28	0.10	0.03
H$\times$M	1.34	0.96	1.05
H$\times$H (very high-risk)	17.19	25.29	14.78
**Total**	100.00	100.00	100.00

**Fig. 3:**
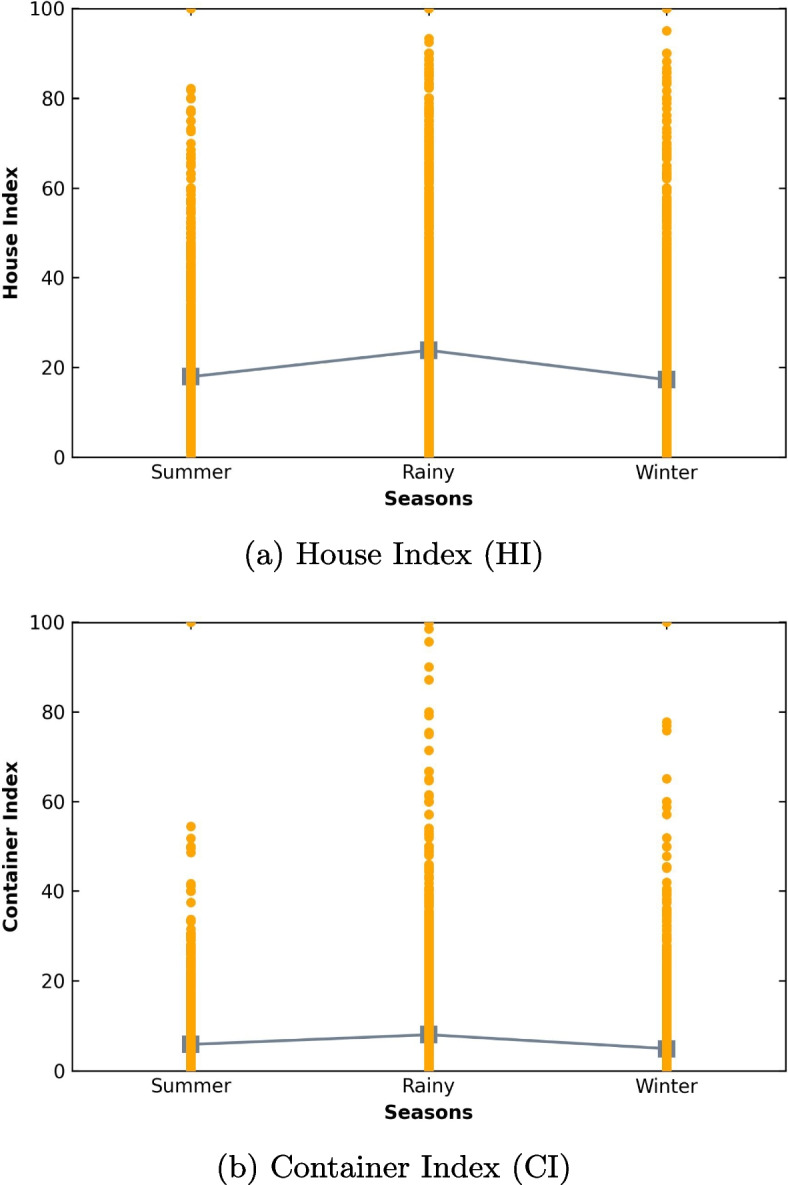
Larval indices of villages across seasons

Figure 3a displays the House Index (HI) of various villages during three seasons: summer, rainy, and winter. The mean HI during each season was 17.92, 23.79, and 17.24, respectively. A one-way ANOVA revealed that HI exhibited a statistically significant difference across seasons (F(2,9907) = 92.57, p < 0.05). Pairwise comparisons with Bonferroni indicated that HI during the rainy season (mean = 23.79, SD = 24.46) was significantly higher than during the summer (mean = 17.92, SD = 20.94) and winter (mean = 17.24, SD = 22.17) seasons. There was no statistically significant difference in HI between the summer and winter seasons. Specifically, the HI during the rainy season was significantly higher than that during the summer and winter seasons, with a significance level of 0.05.

Figure 3b shows the Container Index (CI) of several villages during three seasons: summer, rainy, and winter. The mean CI during each season was 5.87, 8.04, and 4.93, respectively. A one-way ANOVA showed that there was a statistically significant difference in CI among the three seasons (F(2,9907) = 93.89, p < 0.05). Pairwise comparisons with Bonferroni indicated that CI during the rainy season (mean = 8.04, SD = 12.25) was significantly higher than during the summer (mean = 5.87, SD = 8.16) and winter (mean = 4.93, SD = 7.75) seasons. Additionally, CI in the summer was significantly higher than during the winter seasons. Specifically, the CI was highest during the rainy season, followed by the summer season, and was the lowest during the winter season. These differences were statistically significant at a significance level of 0.05.

**Fig. 4:**
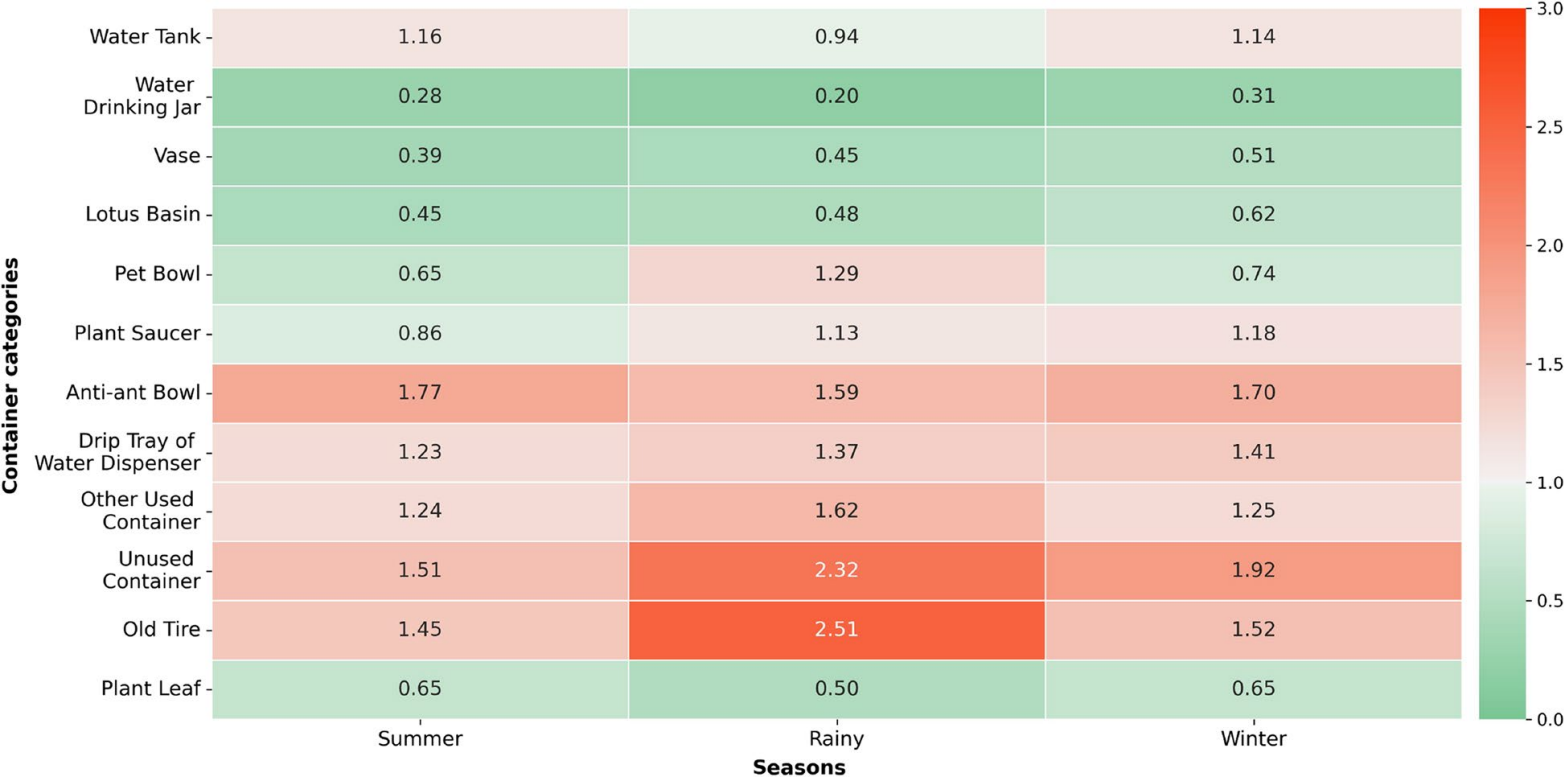
Seasonal breeding preference ratio of wet containers

In addition, the study assessed the essentiality of each container category as breeding habitats for *Aedes* mosquitoes across three seasons using the breeding preference ratio (BPR). Figure 4 shows the seasonal BPR of each container category, indicating their varying degrees of essentiality. Unused containers, old tires and anti-ant bowls were consistently considered essential habitats in all seasons, with a BPR ranging from 1.45 to 2.51. The next most essential habitats were other used containers and drip trays of water dispensers, with an average BPR of 1.37, and 1.34, respectively. Pet bowls were only important breeding habitats during the rainy season, with a BPR of 1.29. Plant saucers were also significant habitats for the rainy and winter seasons, while water tanks were for the summer and winter.

### Village category perspective on very high-risk villages

**Fig. 5:**
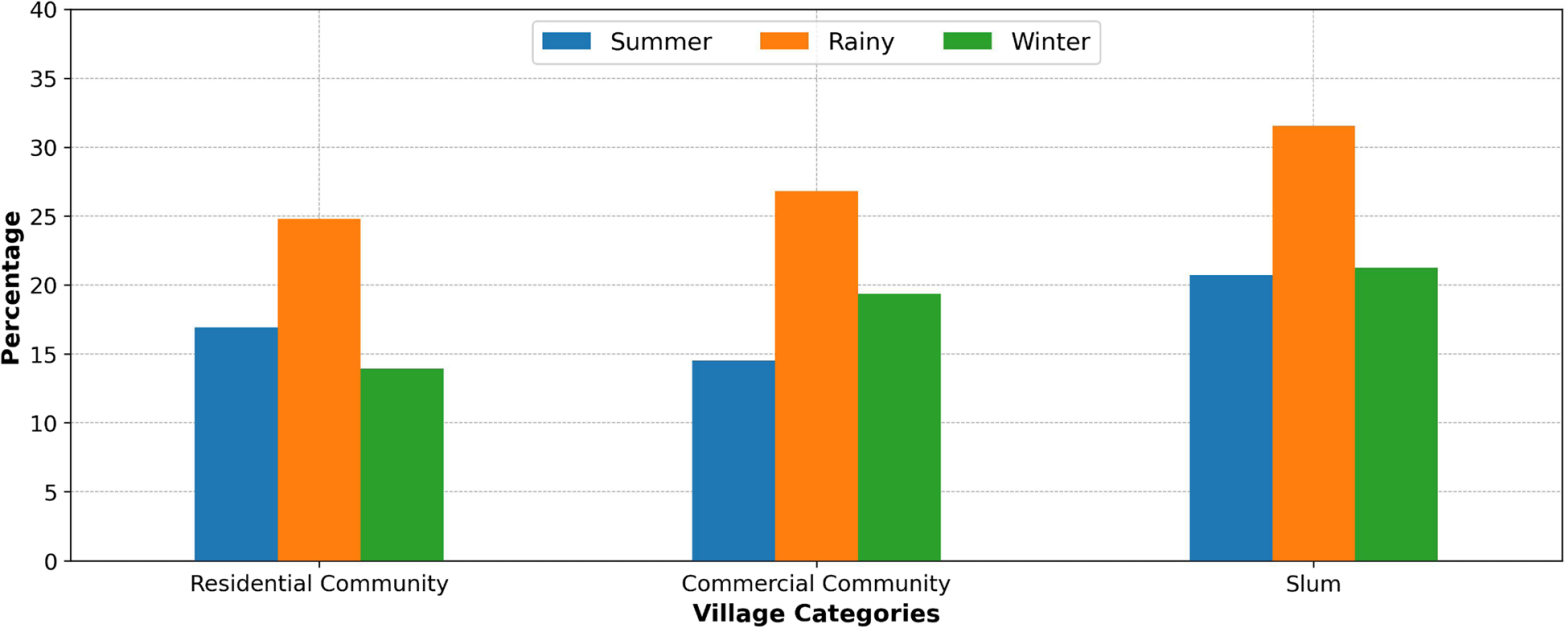
Seasonal percentage of very high-risk villages by categories

The very high-risk villages were explored by measuring their percentages relative to the total number of inspected villages in three village categories (residential communities, commercial communities, and slums) and three seasons. As depicted in Fig. 5, the results indicated that very high-risk villages were predominantly found in slums (from 20.70% to 31.54%) during all seasons, followed by commercial communities (from 14.50% to 26.82%) and then residential communities (from 13.93% to 24.80%). Moreover, a significant proportion of very high-risk villages was found during the rainy season across all village categories.

**Fig. 6:**
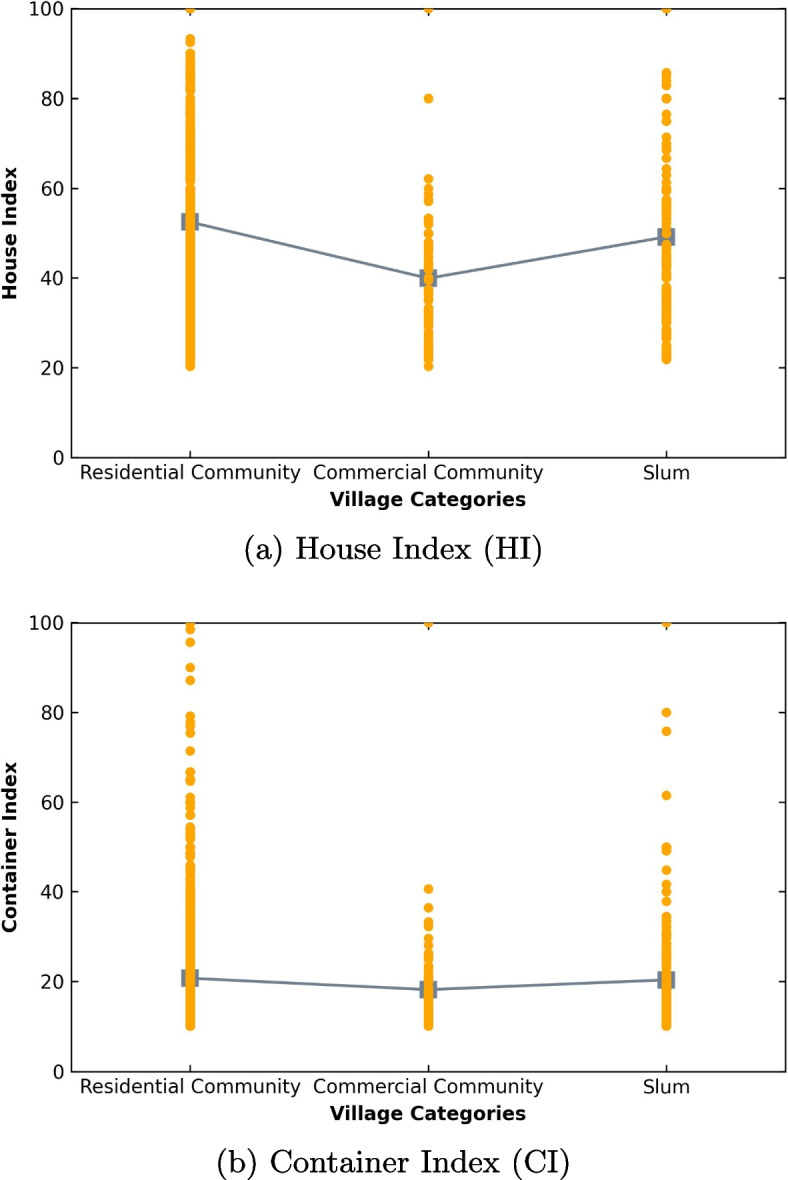
Larval indices of very high-risk villages across village categories

Figure 6a illustrates the House Index (HI) of the very high-risk villages per different categories of villages. The average HI for each village category (residential communities, commercial communities, and slums) was 52.47, 39.93, and 49.17, respectively. A one-way ANOVA was performed to analyze the data, demonstrating a statistically significant difference in HI across all village categories (F(2, 2028) = 16.68, p < 0.05). Bonferroni pairwise comparisons revealed that HI for commercial communities (mean = 39.93, SD = 16.35) was significantly lower than that for residential communities (mean = 52.47, SD = 22.82) and slums (mean = 49.17, SD = 23.15). However, there was no significant difference between HI in residential communities and slums. Specifically, the HI for residential communities and slums was significantly higher than that for commercial communities, indicating that larvae or pupae were present in many houses throughout residential communities and slums.

Figure 6b shows the Container Index (CI) of the very high-risk villages by different categories of villages. The average CI for each village category (residential communities, commercial communities, and slums) was 20.71, 18.17, and 20.34, respectively. A one-way ANOVA indicated that there was no statistically significant difference in CI among the three village categories (F(2, 2028) = 1.37, p = 0.25): residential communities (mean = 20.71, SD = 15.79), commercial communities (mean = 18.17, SD = 12.95), and slums (mean = 20.34, SD = 14.06). Specifically, the risk of dengue transmission as indicated by the CI was similar across different categories of villages among the very high-risk villages.

**Fig. 7:**
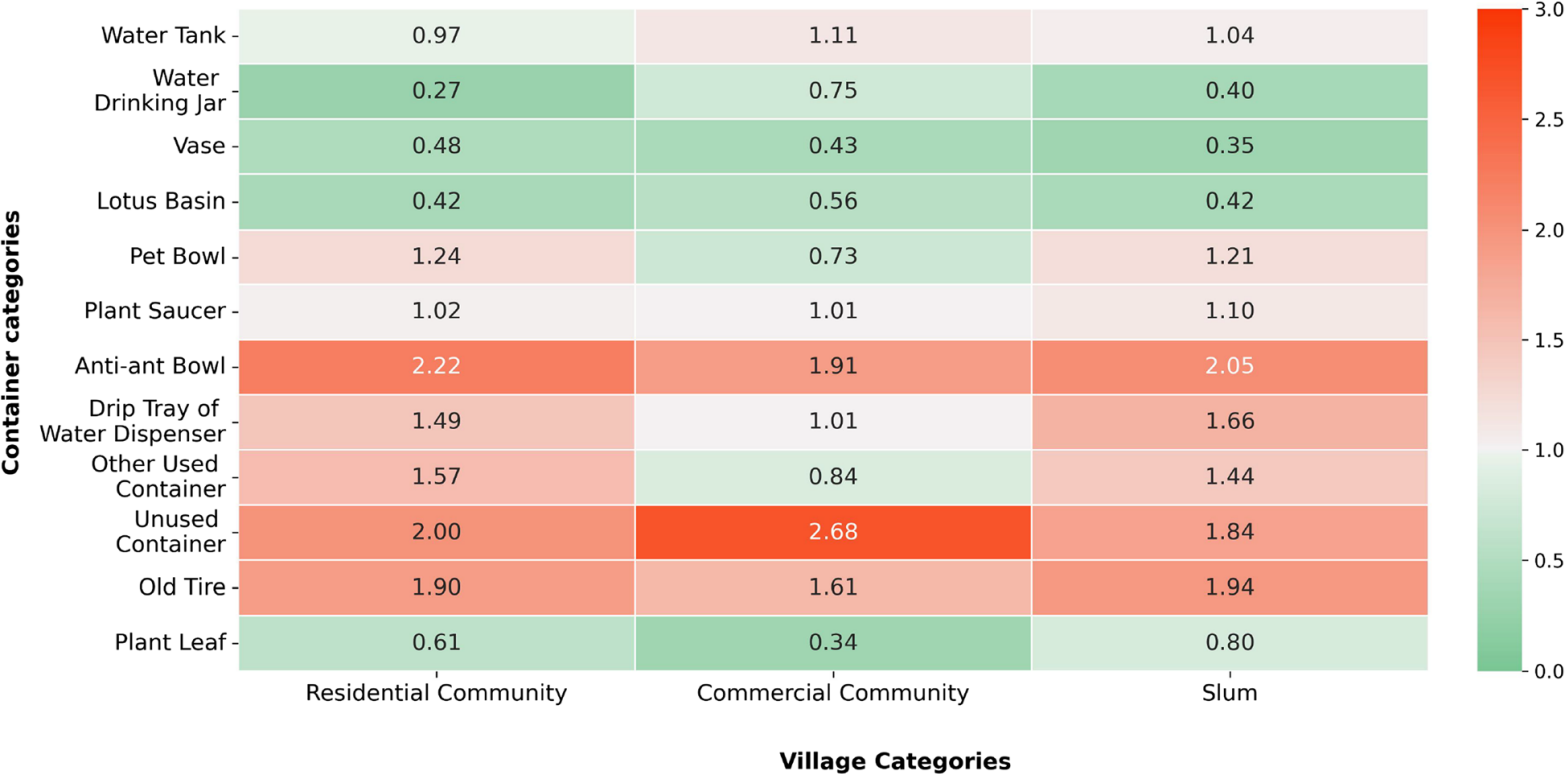
BPR of container habitats in very high-risk villages across village categories

In addition, Fig. 7 illustrates the BPR (Breeding Preference Ratio) of each container category across different village categories. Residential communities and slums had a greater variety of essential breeding habitats compared to commercial communities. Particularly, in commercial communities, essential habitats included unused containers, anti-ant bowls, and old tires, with BPR values of 2.68, 1.91, and 1.61, respectively. On the other hand, residential communities and slums shared several essential habitats, such as anti-ant bowls, unused containers, old tires, drip trays of water dispensers, other used containers, and pet bowls, with BPR values ranging from 1.21 to 2.22.

### Cultural region perspective on very high-risk villages

**Fig. 8:**
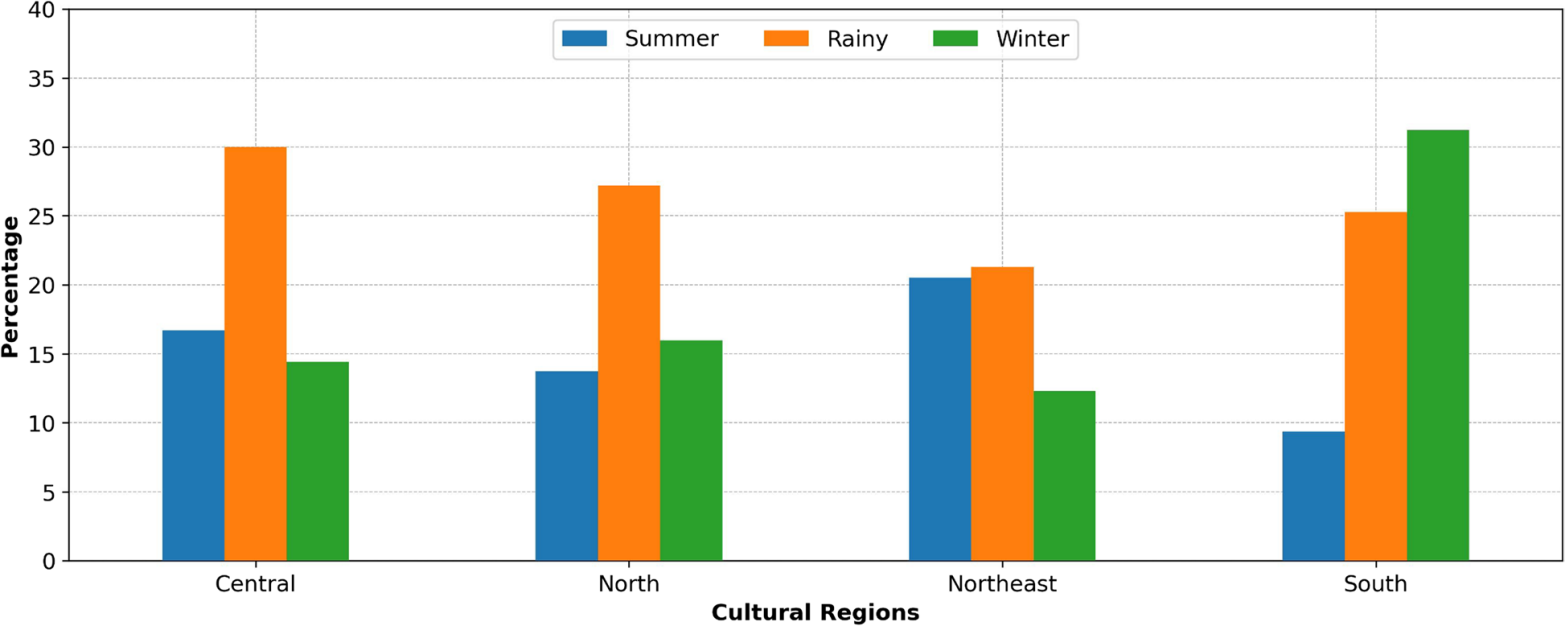
Regional and seasonal percentage of very high-risk villages

**Fig. 9:**
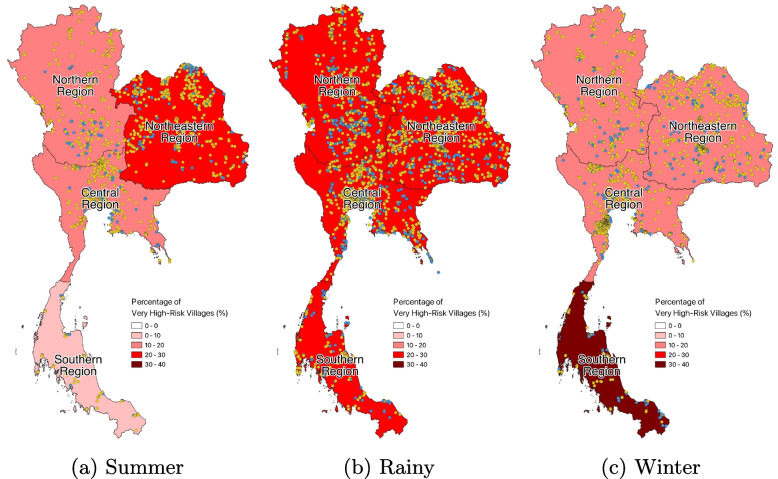
Map of regional and seasonal percentage of very high-risk villages with locations (The blue markers represent inspected villages with a very high-risk level (HI over 20 and CI over 10), while the yellow markers represent the remaining inspected villages)

The very high-risk villages were investigated by measuring their percentages in relation to the total number of inspected villages across four cultural regions (Central, North, Northeast, and South) and three seasons. The findings, illustrated in Figs. 8 and 9, indicated the presence of very high-risk villages in all regions and seasons, ranging from 9% to 31%. The highest incidence of very high-risk villages occurred during the rainy season in all regions, with over 20% prevalence. The Southern region had also a higher proportion of very high-risk villages (over 30%) during the winter seasons. In addition, the Northeastern region had a prevalence of very high-risk villages (about 20%) during the summer season.

**Fig. 10:**
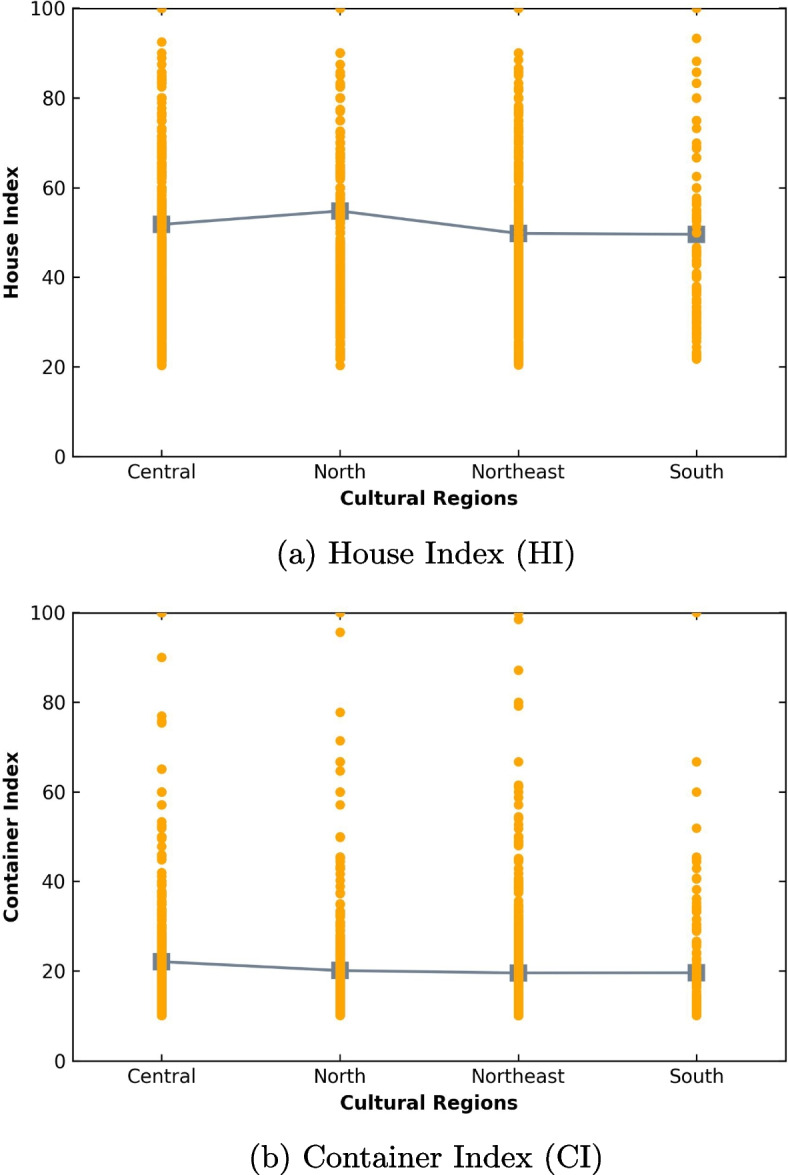
Larval indices of very high-risk villages across regions

Figure 10a illustrates the House Index (HI) of the very high-risk villages per different regions. The average HI for each region (Central, North, Northeast, and South) was 51.78, 54.79, 49.77, and 49.59, respectively. The results of a one-way ANOVA showed a statistically significant difference in HI among the four cultural regions (F(3,2027) = 4.77, p < 0.05). Bonferroni pairwise comparisons revealed a significant difference only between the Northern region (mean = 54.79, SD = 23.25) and the Northeastern region (mean = 49.77, SD = 21.08). No significant difference was found between any other two regions. The mean and standard deviation for the Central region were (mean = 51.78, SD = 23.72), and for the South region were (mean = 49.59, SD = 23.79).

Figure 10b shows the Container Index (CI) of the very high-risk villages by different regions. The average CI for each region (Central, North, Northeast, and South) was 22.06, 20.08, 19.55, and 19.59, respectively. A one-way ANOVA revealed a statistically significant difference in CI among the four cultural regions (F(3,2027) = 3.64, p < 0.05). Bonferroni pairwise comparisons indicated a significant difference only between the Central region (mean = 22.06, SD = 19.57) and the Northeastern region (mean = 19.55, SD = 12.93). No significant difference was found between any other pair of regions. The mean and standard deviation for the Northern region were (mean = 20.08, SD = 12.19), and for the Southern region were (mean = 19.59, SD = 12.86).

**Fig. 11:**
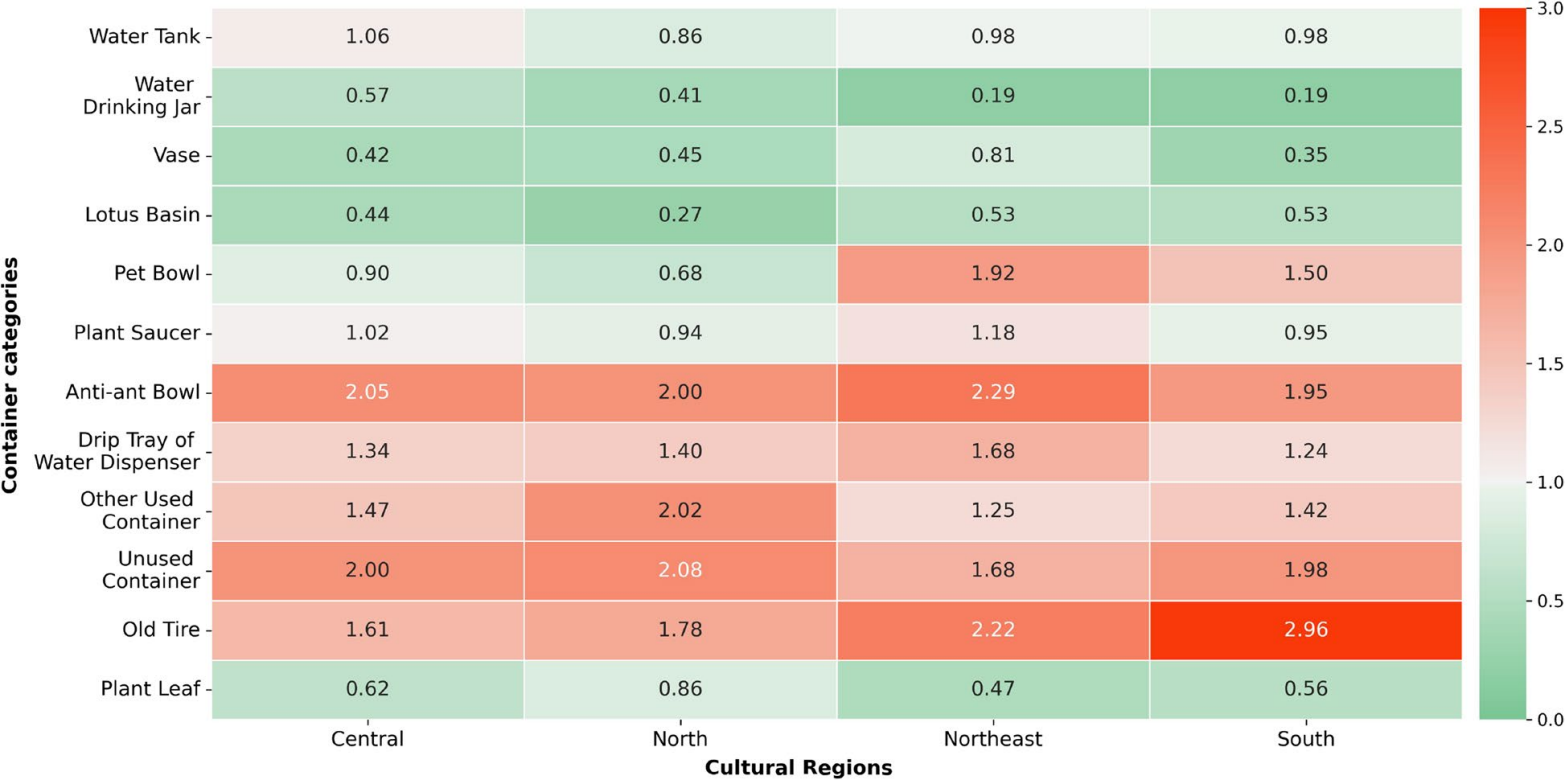
BPR of container habitats in very high-risk villages across regions

Furthermore, Fig. 11 depicts the BPR (Breeding Preference Ratio) of each container category across different cultural regions. All regions had similar essential habitats, with BPR values ranging from 1.25 to 2.96. These included unused containers, old tires, anti-ant bowls, other used containers, and drip trays of water dispensers. Additionally, pet bowls were deemed essential habitats in the Northeast and Southern regions with BPR values of 1.92 and 1.50, respectively. Plant saucers were only observed for the Northeast region with a BPR of 1.18.

## Discussion

The data used in this study was collected from long-term larval surveys carried out nationwide by public health officials. However, the study was limited to the number of wet containers that were inspected and found to be infested with larvae or pupae, without taking into account the density of the infestations. Additionally, the locations of the containers (indoor or outdoor) were not considered due to conflicting definitions and implementation guidelines. Nevertheless, the abundance of long-term larval survey data provided a promising opportunity to precisely identify the villages and breeding habitats that are vulnerable to dengue transmission.

Water containers, such as water tanks, pet bowls, and plant saucers, are ubiquitous in Thai culture. However, they can create breeding grounds for *Aedes* mosquitoes. Villages that harbor water containers may become hotspots for dengue transmission. The study found that villages with varying levels of risk for dengue transmission were present throughout the seasons, ranging from safe to very high-risk. A percentage of villages, ranging from 19.10% to 31.18%, were found to be free from dengue transmission, with the highest percentage observed during the winter season. Conversely, critical hotspots, i.e., the very high-risk villages, had a proportion of 14.78% to 25.29%, with the highest percentage observed during the rainy season. The study also found that House Index (HI) and Container Index (CI) were both significantly higher nationwide during the rainy season than in the summer and winter seasons. Additionally, the CI was significantly higher in the summer than in the winter. These findings indicated that rainfall and temperature are important factors in the breeding of mosquitoes. Similar findings were found in previous studies [32, 33]. Furthermore, climate change, which has made Thailand more susceptible to heavy rainfall, off-season precipitation, and rising temperatures, may be responsible for the presence of very high-risk villages throughout the year.

In addition, the study revealed that slums and residential communities were at a greater risk of dengue transmission, with a higher HI compared to the commercial communities. The vulnerability of these communities may be attributed to factors such as overcrowding, disorganization, and poor management, with human behaviour being the primary contributor [1, 34]. This includes indiscriminate disposal of waste and unused containers, as well as neglecting to remove stagnant water from containers, which create optimal breeding sites for mosquitoes. These factors heighten the prevalence of dengue in slums and residential areas, emphasizing the urgent need for targeted interventions to prevent and manage outbreaks in these communities.

Moreover, the study indicated that villages with a very high-risk of dengue were present throughout the year in all regions. The proportion of such villages was highest during the rainy season in each region. The Southern region had a greater proportion of very high-risk villages during both rainy and winter seasons, as the region experienced rainfall for approximately six months a year during both seasons. The study also found that the Northern region had a considerably higher HI compared to the other regions, while the Central region had a significantly higher CI than the Northern and Northeast regions. These regional differences in dengue risk factors could be influenced by variations in local cultural practices and behaviours. Similar results were found in previous studies [35, 36].

Furthermore, the study identified that all container categories could potentially serve as breeding habitats for mosquitoes. Similar results were found in previous studies [12, 37–39]. Specifically, these containers were found to be breeding habitats throughout the year, with varying degrees of importance based on abandonment and the absence of human activity. Abandoned containers such as unused containers and old tires were found to be the most significant breeding habitats for all seasons (especially the rainy season), all village categories, and all cultural regions. Previous studies [12, 39] also found old tires to be essential breeding sites. Surprisingly, containers that were regularly used such as anti-ant bowls, drip trays of water dispensers, and other used containers were also important breeding habitats.

To ensure the elimination of mosquito breeding sites and reduce the risk of dengue transmission, it is essential for individuals to remain vigilant. This can be achieved through initiatives by the community and the government, along with a sustained public awareness campaign and active community engagement to efficiently and permanently eradicate such sites. Enforcing laws or community regulations to ensure the removal of these breeding grounds can also be effective. Regular surveillance by both the Department of Disease Control and local Public Health Officers is crucial for conducting larval surveys and effectively controlling the spread of mosquitoes. By implementing these measures, a higher proportion of villages can become safe and free from the risk of dengue transmission.

To help prevent the transmission of dengue, the DDC recommends that households follow these guidelines [5]:

- cover water tanks tightly with lids or add biological agents to eliminate mosquito larvae and pupae [40, 41]
- change the water in containers such as drip trays of water dispensers and vases every seven days to interrupt the mosquito larvae cycle [41].
- introduce fish that feed on mosquito larvae into lotus basins [41].
- clean and scrub plant saucers to remove mosquito eggs.
- dispose of unused containers and promote proper waste management [39].
- repurpose old tires into useful items.
- improve ventilation and air circulation in the household environment.

## Conclusions

The aim of this study was to evaluate the villages and breeding habitats that pose a risk for dengue transmission by analyzing long-term larval survey data in Thailand. The study found that villages with a very high-risk of dengue were present throughout the year in all regions. The highest proportion of such villages was observed during the rainy season, which was also associated with a significantly higher House Index (HI) and Container Index (CI). Additionally, among the three village categories, slums and residential communities were more vulnerable to dengue transmission, with a significantly higher House Index.

Moreover, the study identified abandoned containers such as unused containers and old tires as the most significant breeding habitats throughout all seasons, all village categories, and all cultural regions. However, it was surprising to find that regularly used containers, such as anti-ant bowls, drip trays of water dispensers, and other used containers, were also significant breeding habitats. It is thus critical for individuals to remain vigilant in eliminating potential breeding sites.

It should be noted that, according to a preliminary analysis, there was a low correlation between the HI (or CI) and dengue cases at the subdistrict level over the next 28 days following the larval survey. While HI or CI alone may not strongly correlate with dengue cases, they still offer valuable insights into potential mosquito breeding sites, essential for implementing targeted preventive measures and mitigating the risk of transmission.

### Supplementary Information

Supplementary Material 1.

## Data Availability

Data is provided within the manuscript or supplementary information files.

## Abbreviations

BP: Breeding Potentiality
BC: Breeding Contribution
BPR: Breeding Preference Ratio
DDC: Department of Disease Control
WHO: World Health Organization
